# The Binary Model of Chronic Diseases Applied to COVID-19

**DOI:** 10.3389/fimmu.2021.716084

**Published:** 2021-09-03

**Authors:** Zeev Elkoshi

**Affiliations:** Research and Development Department, Taro Pharmaceutical Industries Ltd, Haifa, Israel

**Keywords:** COVID-19, SARS-CoV-2, Treg, corticosteroids (CS), JAK inhibitor, statins, rapamycin, co-infection

## Abstract

A binary model for the classification of chronic diseases has formerly been proposed. The model classifies chronic diseases as “high Treg” or “low Treg” diseases according to the extent of regulatory T cells (Treg) activity (frequency or function) observed. The present paper applies this model to severe acute respiratory syndrome coronavirus 2 (SARS*-*CoV*-*2) infection. The model correctly predicts the efficacy or inefficacy of several immune-modulating drugs in the treatment of severe coronavirus disease 2019 (COVID-19) disease. It also correctly predicts the class of pathogens mostly associated with SARS-CoV-2 infection. The clinical implications are the following: (a) any search for new immune-modulating drugs for the treatment of COVID-19 should exclude candidates that do not induce “high Treg” immune reaction or those that do not spare CD8+ T cells; (b) immune-modulating drugs, which are effective against SARS-CoV-2, may not be effective against any variant of the virus that does not induce “low Treg” reaction; (c) any immune-modulating drug, which is effective in treating COVID-19, will also alleviate most coinfections; and (d) severe COVID-19 patients should avoid contact with carriers of “low Treg” pathogens.

## Introduction

In an earlier paper, a binary classification of chronic diseases was proposed (1). Chronic diseases were classified according to the extent of regulatory T cell (Treg) activity, estimated in peripheral blood or within tissues implicated in the disease. Diseases with high Treg activity as a driver of pathogenicity were classified as “high Treg” diseases (most solid cancers, for example). Diseases with low Treg activity as a driver of pathogenicity were classified as “low Treg” diseases (autoimmune diseases, for example). This classification explains the association of particular pathogens with cancer and the association of others with autoimmune diseases. It also explains why certain specific pathogens are involved in coinfections. The effectiveness or ineffectiveness of certain immune-modulating drugs in the treatment of autoimmunity and cancer is also elucidated by this binary model (1). In addition, it explains why “high Treg” inflammation promotes many solid cancers, while “low Treg” inflammation promotes lymphomas (2). Most COVID-19 patients present mild disease with typical symptoms such as fever, cough, and fatigue. The illness however may progress in some patients to a severe condition with acute respiratory distress syndrome which may lead to multiple organ failure and death (3). Data from coronavirus disease 2019 (COVID-19) patients indicated a decrease in Tregs frequency and function, from mild to severe disease (4, 5). A decrease in CD45RA+ Treg cells frequency between mild and severe states was observed in another study, but the total Tregs frequency was maintained (3). One work reports of an increase in Tregs from mild to severe state and a decrease in Tregs from severe to critical state (6). However, the literature, in general, points to an increase in proinflammatory cytokines, which extenuates in severe disease. This so called cytokine storm includes cytokines such as interferon gamma (IFN-γ), interleukin (IL)-1, IL-6, IL-8, IL-12, and transforming growth factor β (TGF-β) and chemokines like C–C motif ligand 2 (CCL2), CXCL9, and CXCL10. Elevated levels of plasma IL-2, IL-7, IL-10, tumor necrosis factor-α (TNF-α), granulocyte-macrophage colony stimulating factor (GM-CSF), macrophage inflammatory protein 1-α (MIP1-α), and MCP-1 have also been reported. This cytokine storm results in a collateral damage, especially to lung tissues, which may progress to a critical condition and death (7). It is clear that COVID-19 may evolve progressively into a proinflammatory “low Treg” state. This is in contrast to cancers like hepatocellular carcinoma that progress from a “low Treg” state to a “high Treg” state (8).

**Figure 1:**
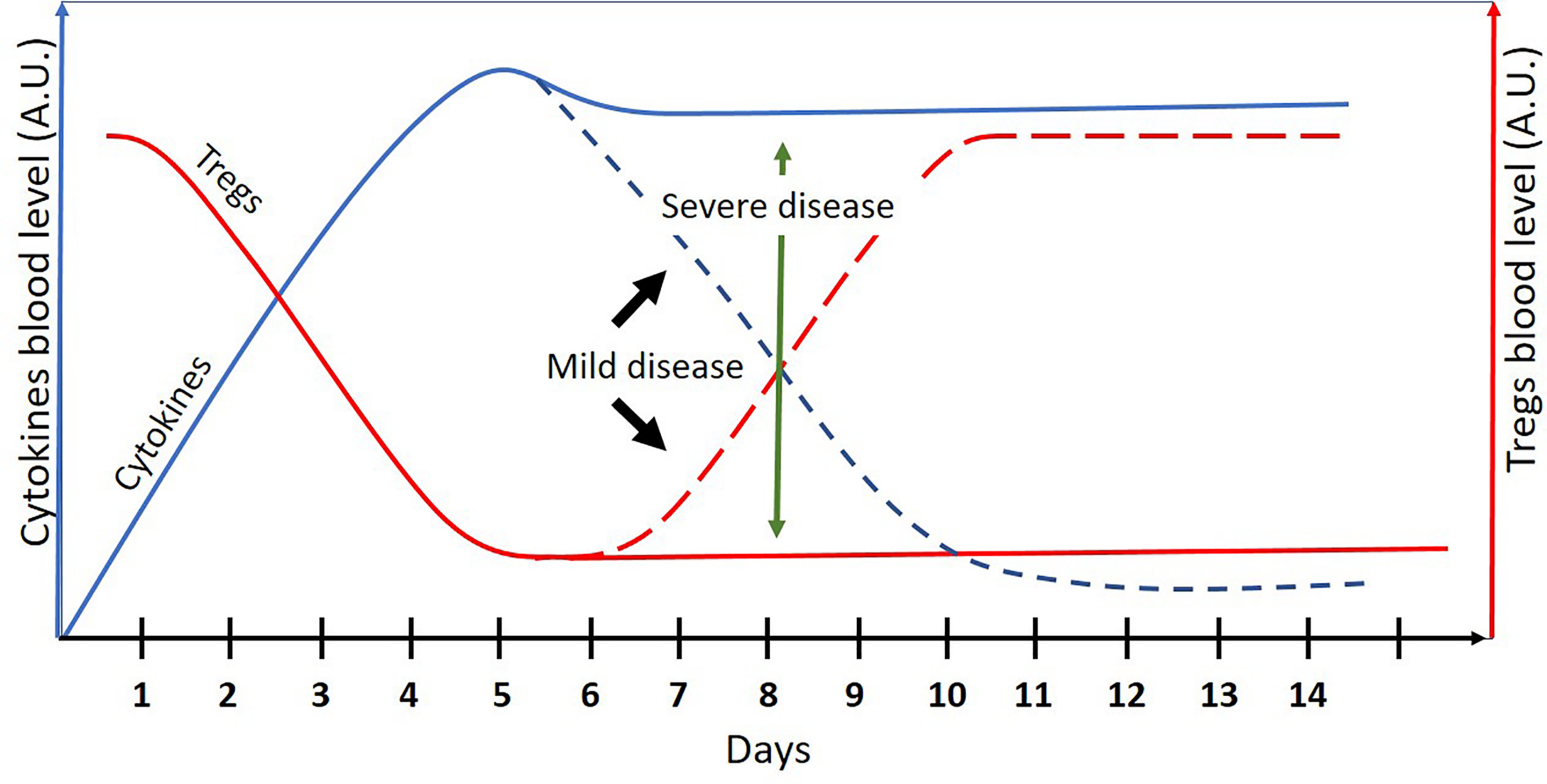
A schematic presentation of cytokines and Treg cells blood levels (arbitrary units) in COVID-19 patients as a function of time (days). The dashed lines represent the mild disease. Mild COVID-19 may be regarded as an acute and self-resolving inflammation. Severe COVID-19 may be regarded as “low Treg” chronic inflammation where the binary model of chronic diseases can be applied.

Mild COVID-19 may be regarded as a self-resolving acute inflammation. Severe COVID-19, on the other hand, is a persistent “chronic” proinflammatory disease, classified as a “low Treg” disease by the binary model of chronic diseases (1) (see **Figure 1**). As such, the binary model may be useful in (a) screening for effective immune-modulating drugs that may improve the outcomes of severe COVID-19 and (b) explaining why some pathogens are more frequently associated with severe COVID-19 while others are not.

## Immune-Modulating Drugs in Severe COVID-19

According to the binary model, drugs that induce Tregs activity (either frequency or function) are expected to have a beneficial effect on severe COVID-19 if they do not hamper the specific antiviral immune reaction.

Specific immunity against severe acute respiratory syndrome coronavirus 2 (SARS*-*CoV*-*2) infection is double arm: humoral antibody response and T-cell response (9), mainly CD8+ T cells (cytotoxic T lymphocytes [CTL]) (10). Immune response to infection is diverse and redundant in order to allow reaction when a certain branch of immunity is hampered (11). For example, CTL robust reaction may cover up for impaired specific antibody reaction in hematological cancer patients with SARS-CoV-2 infection (12).

It is reasonable therefore to assume that Tregs inducers, which suppress the inflammatory response but spare specific CD8+ T-cell anti-CoV-2 activity, will be effective in treating severe COVID-19. Similarly, Tregs inducers that have a direct antiviral activity are expected to be effective in severe COVID-19, as well.

Here are some examples of drugs that conform to these conditions, drugs that do not, and drugs that may affect the corona virus in additional modes of action, which do not involve the immune system:

- Corticosteroids: corticosteroids (CS) have shown to promote Tregs proliferation *in vitro* (13) and *in vivo* (14). Corticosteroids are used for years as immune suppressors in the treatment of autoimmune diseases. Nevertheless, CS upregulated leukotriene B4 receptor 1 (BLT1) expression on effector memory CD8+ T cells and potentiated airway inflammation induced by these cells in mice (15). Moreover, dexamethasone promoted apoptosis of naive and memory CD8+ T cells in a mouse model of herpesvirus infection, but virus-specific CD8+ T cell were preserved (16). In addition, CS did not suppress high-affinity tumor-specific memory CD8+ T cells in a mouse tumor model (even though low-affinity cells were suppressed) (17). Corticosteroids are therefore anticipated to improve the outcomes of severe COVID-19. Indeed, the World Health Organization recommends the use of CS in severe COVID-19 but not in mild disease (see *Discussion*).
- JAK inhibitors: most JAK inhibitors induce an anti-inflammatory effect expressed by increased Tregs activity and/or decreased Th17 activity (18). The effect of JAK inhibitors on CTL may vary from drug to drug. In rheumatoid arthritis patients, tofacitinib did not affect the absolute number of cytotoxic T lymphocytes (CTL) (19). Baricitinib supported CTL activation in severe COVID-19 patients (20). Ruxolitinib, however, suppressed CTL numbers and cytokine-producing capacity in hemophagocytic lymphohistiocytosis mouse model (a cytokine storm disease model) (21). Ruxolitinib also suppressed CTL in mice immunized with ovalbumin (an allergic reaction model) (22). Considering their effect on TCL, tofacitinib and baricitinib are expected to be effective in treating severe COVID-19 while ruxolitinib may not. Recent large clinical studies support the use of tofacitinib and baricitib but not of ruxolitinib in severe COVID-19 patients. In addition, Food and Drug Administration (FDA) issued an authorization for the emergency use of baricitinib in severe COVID-19 patients (see *Discussion*).
- Rapamycin: rapamycin induces Tregs expansion *in vitro* (13) and Tregs expansion and function in diabetic or IPEX patients (23, 24). Mouse models demonstrated a promotion of memory CTL differentiation and function by rapamycin (25–27). By its immune-modulating effects, rapamycin is expected to improve severe COVID-19 outcomes if the drug have no other effects on the virus vitality or life cycle. However mTOR, the mammalian target of rapamycin, is a master regulator of several key cellular processes, including protein nucleotide and lipid synthesis, glutamine metabolism, glycolysis, and autophagy (28). In addition, mTOR lowers the intrinsic barrier to some viral infections (29). The overall effect of rapamycin in COVID-19 is therefore hard to forecast by the binary model alone.
- Statins: Tregs induction—simvastatin and lovastatin induced Tregs proliferation *in vitro* (13) and *in vivo* (30, 31). Atorvastatin and rosuvastatin induced Tregs proliferation *in vivo* (32). Fluvastatin and pravastatin did not promote Tregs expansion *in vitro* (13).
  Effect on CTL—simvastatin promoted the function of CTL within tumor microenvironment (33) and in sepsis mouse model (34). Lovastatin inhibited the proliferation of virus-specific CTL without affecting their cytolytic capacity (35). Atorvastatin induced reduction and exhaustion in CTL in HIV patients, while pravastatin have shown no effect (36). Fluvastatin-pretreated donor cells attenuated CTL function in acute graft-versus-host disease mouse model (37). Rosuvastatin suppressed CTL activity in some subpopulations of healthy subjects (38) and HIV-infected patients (39).

Direct antiviral effect—inhibition or slowing down viral replication is common to several statins, due to their ability of inhibiting cholesterol production, reducing the availability of some isoprenoids that are essential for regulating the virus life cycle and by inhibiting the activity of regulatory proteins related to virus intracellular life cycle (40). Reduction of viral entry into host cells is another antiviral effect of statins (41). A recent work examined the effect of several statin on SARS-Cov-2 cell entry and infection in human respiratory epithelial cell line. Only fluvastatin demonstrated a statistically significant effect on the reduction in infection (41). It should be realized, however, that fluvastatin concentration used in this work, 10 μM (=4.11 μg/ml), is about one order of magnitude higher than the observed maximum blood concentration of the drug (Cmax), at steady state, following 40 mg daily dosing of fluvastatin tablets, to healthy subjects (Cmax = 0.438 μg/ml) (42). At 0.4 μg/ml drug concentration, the effect of fluvastatin on SARS-Cov-2 infectivity in human respiratory epithelial cells is negligible, by a dose–response curve (41). It can be concluded that at therapeutic drug levels, a direct antiviral effect of statins on SARS-Cov-2 infection is not plausible.

Cholesterol synthesis effect*—*compared to healthy subjects, COVID-19 patients present lower levels of serum cholesterol, high-density lipoproteins (HDLs), and low-density lipoproteins (LDL), which correlate with disease severity (43). The inhibitory effect of statins on cholesterol synthesis may protect against COVID-19 infection. Simvastatin has been shown to reduce polymorphonuclear cells membrane cholesterol and affect membrane cell fluidity (44). It may be hypothesized that these membrane changes by simvastatin reduce SARS-CoV-2 cell entry.

**Table 1** summarizes the effect of several statins on Tregs and CD8+ T cells.

**Table 1:** The effect of several statins on Tregs and CD8^+^ T cells.

	Tregs (Ref.)	CD8^+^ T cells (Ref.)
Simvastatin	↑ (13, 30, 31)	↑ (33, 34)
Lovastatin	↑ (13, 30, 31)	↔ (35)
Atorvastatin	↑ (32)	↓ (36)
Fluvastatin	↔ (13)	↓ (37)
Rosuvastatin	↑ (32)	↓ (38, 39)
Pravastatin	↔ (13)	↔ (36)

↑ = An increase, ↓ = A decrease, ↔ = No effect.

Considering their effect on Tregs and CTL, simvastatin and lovastatin are expected to be the most effective in severe COVID-19, while fluvastatin is expected be the least effective if statins have no other effects on the pathology of the disease. It should be added that the data in **Table 1** has not been evaluated under SARS-CoV-2 infection conditions, which may affect their validity in any assessment related to COVID-19.

## Coinfections in Severe COVID-19 Patients

According to the binary model, “low Treg” disease such as severe COVID-19 is expected to be associated with “low Treg” pathogens.

A review of coinfections observed in hospitalized COVID-19 patients indicate the following bacterial infections (in a descending order of their prevalence) (45): *Mycoplasma pneumoniae*, *Pseudomonas aeruginosa*, *Haemophilus influenza*, *Klebsiella pneumoniae*, *Enterobacter* spp., *Chlamydia* spp., *Acinetobacter baumannii*, *Serratia marcescens*, methicillin-resistant *Staphylococcus aureus*, and *Enterococcus faecium*.

Seven out of these 10 bacteria have demonstrated a “low Treg” reaction (either Tregs reduction or Th17 expansion or both): *M. pneumonia* (46), *P. aeruginosa* (1), *H. influenza* (47), *K. pneumoniae* (48), *Chlamydia* (1), *A. baumannii* (49), and methicillin-resistant *S. aureus* (50). *Enterococcus faecium*, the only species in the list that exhibits a “high Treg” reaction (51), is the last in the list and has the lowest prevalence. No data regarding the effect of *S. marcescens* on the immune adaptive system could be found. No data could be found regarding the effect of the *Enterobacter* genus. However, *E. coli*, a member of the *Enterobacteriaceae* family, induced a Th17 reaction (52). Another member of the *Enterobacteriaceae* family, *Citrobacter rodentium*, presented a strong Th17 reaction during gut infection, but this reaction required the presence of Treg cells (53). **Table 2** summarizes these observations.

**Table 2:** Bacterial co-infections in COVID-19 patients (in a descending order of their prevalence) (45) and Treg reaction to the bacteria in the absence of SARS-CoV2.

Bacterium	Treg reaction in the absence of SARS-CoV2 infection	Reference
*Mycoplasma pneumoniae*	Low	(46)
*Pseudomonas aeruginosa*	Low	(1)
*Haemophilus influenza*	Low	(47)
*Klebsiella pneumoniae*	Low	(48)
*Enterobacter* spp.	Not available	–
*Chlamydia* spp.	Low	(1)
*Acinetobacter baumannii*	Low	(49)
*Serratia marcescens*	Not available	–
Methicillin-resistant *Staphylococcus aureus*	Low	(50)
*Enterococcus faecium*	High	(51)

The following 12 viruses are associated with SARS-CoV-2 infection (in a descending order of their prevalence) (45): respiratory syncytial virus (RSV), influenza A virus, rhinovirus, enterovirus, influenza B virus, parainfluenzae, other corona viridae, adenovirus, human metapneumovirus(hMBV), Epstein–Barr virus (EBV), Coxsackievirus (COX), and cytomegalovirus (CMV).

Ten out of these 12 viruses have shown to elicit “low Treg” immune response (Th17 expansion or enhanced Th17 cytokine signature):RSV (54), influenza A virus (55), enterovirus (56), influenza B virus, parainfluenzae (57), Middle East respiratory syndrome coronavirus (MERS-CoV) (58), adenovirus (59), hMPV (60), COX (61), and CMV (62).

*EBV* may induce either “low Treg” or “high Treg” reaction, depending on the disease or infection (1). **Table 3** summarizes these observations.

**Table 3:** Viral coinfections in COVID-19 patients (in a descending order of their prevalence) (45) and Treg reaction to the viruses in the absence of SARS-CoV2.

Virus	Treg reaction in the absence of SAS-CoV2 infection	Reference
Respiratory Syncytial Virus	Low	(54)
Influenza A virus	Low	(55)
Rhinovirus	Not available	–
Enterovirus	Low	(56)
Influenza B virus	Low	(57)
Parainfluenzae	Low	(57)
MERS-CoV	Low	(58)
Adenovirus	Low	(59)
Human metapneumovirus	Low	(60)
Epstein–Barr virus	Low/high (depending on the disease)	(1)
Coxsackievirus	Low	(62)
Cytomegalovirus	Low	(61)

## Discussion

As mentioned above, mild COVID-19 may be regarded as an acute self-resolving inflammation, while a severe disease may be viewed as a chronic “low Treg” disease (**Figure 1**). According to the binary model of chronic diseases, Treg inducers may be effective in chronic “low Treg” diseases such as autoimmune diseases, “low Treg” infections, asthma (1), and lymphomas (2); however, their use is not recommended in acute inflammations. Immune-modulating drugs may disturb the delicate balance between the pro- and anti-inflammatory arms of the immune system, needed in acute inflammations for a successful pathogen elimination without causing an excessive collateral damage.

In order to allow its use for assessing the efficacy of drugs in COVID-19, the original binary model has been modified to include the effect of the drugs on the specific immunity against the virus. Several works demonstrate the importance of specific CD8+ T cells in the pathology of COVID-19. For example, impaired CTL response has been reported in COVID-19 elderly patients (63). On the other hand, strong and broad memory CD8+ (and CD4+) T cells were detected in convalescent population following COVID-19 (64). The induction of virus-specific CTL in mice conferred substantial protection against lethal dose of SARS-CoV-2 challenge (65).

Corticosteroids in general are Treg promoters (1). As stated above, dexamethasone anti-inflammatory effect spares the herpesvirus-specific CTL reaction. Hence, dexamethasone is a good candidate for treating severe COVID-19 but not mild COVID-19. Indeed, a large clinical study investigating the use of dexamethasone in hospitalized COVID-19 patients concluded that “the use of dexamethasone resulted in lower 28-day mortality among those who were receiving either invasive mechanical ventilation or oxygen alone at randomization but not among those receiving no respiratory support” (66). Based on clinical data, the World Health Organization recommends the use of oral CS for the treatment of patients with severe and critical COVID-19 but not in the treatment of patients with non-severe COVID-19 (67).

As for JAK inhibitors, the model predicts the efficacy of tofacitinib and baricitinib in the treatment of severe COVID-19. Ruxolitinib is expected to be less effective due to its suppressive effect on CTL number and function (21, 22). Small-scale clinical studies have demonstrated the positive effect of baricitinib and ruxolitinib on the clinical outcomes of COVID-19 (68). However, a recent placebo-controlled Phase III clinical trial of ruxolitinib in COVID-19 patients has failed to meet its primary endpoint of reducing the number of hospitalized COVID-19 patients who experienced severe complications (69). Another Phase III clinical trial (the DEVENT study) which evaluated the efficacy and safety of ruxolitinib 5 mg and 15 mg plus standard of care (SOC) compared to SOC plus placebo in patients with severe COVID-19, “did not meet its primary endpoint—mortality due to any cause through day 29—adjusted for ARDS severity between the two treatment arms versus placebo” (70). On the other hand, a Phase III clinical trial demonstrated that “baricitinib plus remdesivir was superior to remdesivir alone in reducing recovery time and accelerating improvement in clinical status among patients with COVID-19, notably among those receiving high-flow oxygen or noninvasive ventilation” (71). In addition, a Phase III placebo controlled clinical study of baricitinib in 1,525 hospitalized adults with COVID-19 receiving SOC have demonstrated that “treatment with baricitinib in addition to SOC (predominantly dexamethasone) significantly reduced mortality with a similar safety profile between groups of hospitalized COVID-19 participants.” On the other hand, reduction in disease progression did not achieve statistical significance in this study (72). Accordingly, on November 19, 2020, FDA issued an Emergency Use Authorization (EUA) for the emergency use of baricitinib (Olumiant), in combination with remdesivir (Veklury), for the treatment of severe COVID-19 patient. On July 28, 2021, FDA renewed this EUA. At the same date, FDA issued an EUA for the emergency use of (stand-alone) baricitinib for the treatment of severe COVID-19 patients (73).

A recent placebo controlled study in 289 COVID-19 Brazilian patients hospitalized with pneumonia, have shown that “tofacitinib led to a lower risk of death or respiratory failure through day 28 than placebo” (74).

The binary model is useful in predicting the efficacy of drugs that modulate the immune response. However, when immune-modulating drugs affect the disease by additional mechanisms that do not engage the immune system, the model may fail. As presented above, statins may affect SARS-CoV-2 infection by immune-related and immune-unrelated modes of action. Therefore, the binary model may fail to explain their effect in COVID-19. Here is an example: atorvastatin or simvastatin routine use by a group of cardiovascular patients seemed to have a protective role against SARS-CoV-2. All-cause mortality among COVID-19 patients in this group was lower, relative to a control group of COVID-19 patients with cardiovascular background who did not take statins. On the other hand, rosuvastatin or pravastatin use by COVID-19 patients with a cardiovascular history was associated with a higher all-cause mortality compared to the control group (75). About 85% of the patients in the first group used atorvastatin and about 87% of the patients in the second group used rosuvastatin. Hence, atorvastatin seems to have a protective effect on COVID-19 outcomes, while rosuvastatin worsen COVID-19 outcomes in patients with cardiovascular history. This favorable effect of atorvastatin over rosuvastatin cannot be explained by their immune-modulating impacts as presented in **Table 1**. Since direct antiviral effects are generally low at therapeutic blood levels of statins, as explained before, different effect by each drug on epithelial cell membrane is a possible cause for the observed difference in their impact on the disease outcomes. Evidently, the binary model alone is not sufficient for elucidating the different effects of these two statins on the prognosis of severe COVID-19.

Rapamycin is another example where the binary model alone cannot foretell the drug efficacy or inefficacy in the treatment of COVID-19 due to possible immune-unrelated effects.

According to the binary model, as a “low Treg” disease, severe COVID-19 is expected to be associated mainly with “low Treg” coinfections.

Indeed, at least 7 out of 10 bacteria and 10 out of 11 viruses associated with COVID-19 are “low Treg” pathogens, as aforementioned. No “high Treg” virus is reported among viruses prevalent in COVID-19. The only “high Treg” bacteria reported (*Enterococcus faecium*) has the lowest prevalence in the list of bacterial coinfections in COVID-19. It is seen that coinfections associated with COVID-19 are almost exclusively “low Treg” infections, as asserted by the binary model.

## Clinical Implications

There are several clinical implications of the findings of this work:

(a) Drugs that do not induce “high Treg” immune reaction or do not spare CD8+ T cells should be excluded from any search for new immune-modulating drugs for the treatment of severe COVID-19.
(b) Immune-modulating drugs, which are effective against SARS-CoV-2, may not be effective against any variant of the virus that does not induce “low Treg” reaction.
(c) In the case of severe COVID-19 associated with coinfection, any immune-modulating drug that is effective against SARS-CoV-2 is expected to alleviate the coinfection as well.
(d) Severe COVID-19 patients should avoid contact with carriers of “low Treg” pathogens (whether or not these pathogens are known to be highly associated with the disease).

## Conclusions

The binary model of chronic diseases correctly predicts the efficacy of several immune-modulating drugs in treating severe COVID-19. It also correctly predicts the class of pathogens mostly associated with COVID-19. These observations have important clinical implications.

## Data Availability Statement

The original contributions presented in the study are included in the article/supplementary material. Further inquiries can be directed to the corresponding author.

## Author Contributions

The author confirms being the sole contributor of this work and has approved it for publication.

## Author Disclaimer

The views and opinions expressed, and/or conclusions drawn, in this article are those of the author and do not necessarily reflect those of Taro Pharmaceutical Industries Ltd., its affiliates, directors, or employees.

## Conflict of Interest

ZE is employed by Taro Pharmaceutical Industries Ltd.

## Publisher’s Note

All claims expressed in this article are solely those of the authors and do not necessarily represent those of their affiliated organizations, or those of the publisher, the editors and the reviewers. Any product that may be evaluated in this article, or claim that may be made by its manufacturer, is not guaranteed or endorsed by the publisher.
